# Supplementary material for: Causal Relationship between Aging and Anorexia Nervosa: A White-Matter-Microstructure-Mediated Mendelian Randomization Analysis
Source: Biomedicines. 2024 Aug 16;12(8):1874. doi: 10.3390/biomedicines12081874 (PMC11351342; doi:10.3390/biomedicines12081874)
Supplement: Supplementary file 1 [file biomedicines-12-01874-s001.zip › Supplementary Figure/Figure S4/Singe SNP plot of MR analysis for TL to ubm-b-1497.pdf]

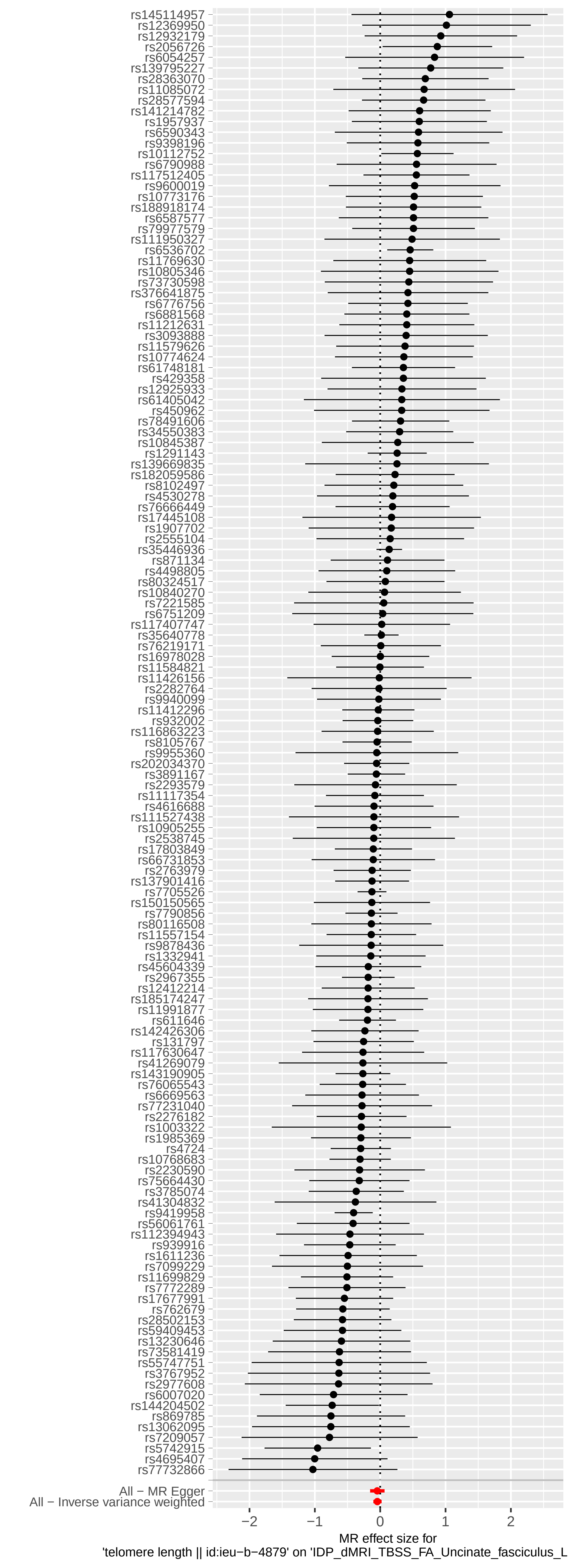
rs145114957
rs12369950
rs12932179
rs2056726
rs6054257
rs139795227
rs28363070
rs11085072
rs28577594
rs141214782
rs1957937
rs6590343
rs9398196
rs10112752
rs6790988
rs117512405
rs9600019
rs10773176
rs188918174
rs6587577
rs79977579
rs111950327
rs6536702
rs11769630
rs10805346
rs73730598
rs376641875
rs6776756
rs6881568
rs11212631
rs3093888
rs11579626
rs10774624
rs61748181
rs429358
rs12925933
rs61405042
rs450962
rs78491606
rs34550383
rs10845387
rs1291143
rs139669835
rs182059586
rs8102497
rs4530278
rs76666449
rs17445108
rs1907702
rs2555104
rs35446936
rs871134
rs4498805
rs80324517
rs10840270
rs7221585
rs6751209
rs117407747
rs35640778
rs76219171
rs16978028
rs11584821
rs11426156
rs2282764
rs9940099
rs11412296
rs932002
rs116863223
rs8105767
rs9955360
rs202034370
rs3891167
rs2293579
rs11117354
rs4616688
rs111527438
rs10905255
rs2538745
rs17803849
rs66731853
rs2763979
rs137901416
rs7705526
rs150150565
rs7790856
rs80116508
rs11557154
rs9878436
rs1332941
rs45604339
rs2967355
rs12412214
rs185174247
rs11991877
rs611646
rs142426306
rs131797
rs117630647
rs41269079
rs143190905
rs76065543
rs6669563
rs77231040
rs2276182
rs1003322
rs1985369
rs4724
rs10768683
rs2230590
rs75664430
rs3785074
rs41304832
rs9419958
rs56061761
rs112394943
rs939916
rs1611236
rs7099229
rs11699829
rs7772289
rs17677991
rs762679
rs28502153
rs59409453
rs13230646
rs73581419
rs55747751
rs3767952
rs2977608
rs6007020
rs144204502
rs869785
rs13062095
rs7209057
rs5742915
rs4695407
rs77732866
All − MR Egger
All − Inverse variance weighted
−2
−1
0
1
2
MR effect size for
'telomere length || id:ieu−b−4879' on 'IDP_dMRI_TBSS_FA_Uncinate_fasciculus_L
